# Perchlozone Resistance in Clinical Isolates of *Mycobacterium tuberculosis*

**DOI:** 10.3390/antibiotics12030590

**Published:** 2023-03-15

**Authors:** Anastasia Ushtanit, Yulia Mikhailova, Ludmila Krylova, Dmitry Grigorash, Marina Makarova, Svetlana Safonova, Danila Zimenkov

**Affiliations:** 1 Center for Precision Genome Editing and Genetic Technologies for Biomedicine, Engelhardt Institute of Molecular Biology, Russian Academy of Sciences, 119991 Moscow, Russia; 2 The Moscow Research and Clinical Center for Tuberculosis Control, Moscow Government Health Department, 107014 Moscow, Russia; 3 Join-Stock Company «Pharmasyntez», 664040 Irkutsk, Russia

**Keywords:** tuberculosis, drug resistance, molecular determinants, perchlozone, ethionamide, isoniazid, thioacetozone

## Abstract

The emergence of drug-resistant tuberculosis forced the development of new drugs and the screening of more effective or less toxic analogues. Mycolic acid biosynthesis is targeted by several antituberculosis drugs, isoniazid being one of the most important in tuberculosis therapy. Recently, perchlozone, acting on another step in the FAS-II cycle, was officially approved for tuberculosis treatment in the Russian Federation and was included in the Russian national clinical guidelines. Using the serial dilution method on 7H10 agar plates for perchlozone and a Sensititre MYCOTB microdilution plate, we analyzed the phenotypic properties of primary clinical isolates of *M. tuberculosis* and analyzed the molecular determinants of resistance to isoniazid, ethionamide, and perchlozone. We found a wide variation in the MIC of perchlozone from 2 to 64 mg/L, correlating with the overall resistance profile: the MIC was higher for MDR and pre-XDR isolates. The cross-resistance between ethionamide and perchlozone was driven by mutations in the *ethA* gene encoding monooxygenase responsible for the activation of both drugs. The presumably susceptible to perchlozone and wild-type strains had MICs ranging from 2 to 4 mg/L, and the breakpoint was estimated to be 4 or 8 mg/L. In conclusion, susceptibility to perchlozone is retained for a part of the MDR strains, as is susceptibility to ethionamide, providing the possibility of therapy for such cases based on phenotypic or molecular analysis.

## 1. Introduction

Tuberculosis currently remains a major public health problem worldwide. The transmission of drug-resistant strains of *M. tuberculosis* is becoming common in many regions of the world. The numbers of primary multidrug-resistant tuberculosis (MDR) and extensively drug-resistant (XDR) cases are increasing steadily. Chemotherapy regimens for such patients need to be modified in a timely manner to include effective drugs.

Thiosemicarbazones is a group of chemical compounds with mycobactericidal activity, discovered after a systematic investigation of sulfonamides and thiazoles as antituberculosis agents [1]. The best-known representative of this group was thioacetazone, also known as tibione or Tb1, the first synthetic antituberculosis drug, which began to be used in the late 1940s. It was widely tested and used in Europe after World War II, and is still used in the Asia, Africa, and South America regions as a cheap alternative to ethambutol in first-line therapy. The restricted use of tibione is caused by serious adverse reactions such as gastrointestinal, hepatic, renal disorders, and severe skin reactions in HIV-positive patients [2,3].

In the search for less toxic compounds, three isomeric forms of nicotinaldehyde thiosemicarbazone in the alpha, beta, and gamma positions were discovered in 1952 [4]. Beta and gamma forms were less toxic than thioacetazone. However, simultaneously, the high antituberculosis activity and low toxicity of the intermediate product isonicotine hydrazine (isoniazid) was also shown [5]. This led to intensive studies on isoniazid as one of the most effective antituberculosis drugs with bactericidal mode of action, and it is still one of the most important first-line drugs used also for preventive therapy [6,7].

The emergence of drug-resistant tuberculosis has pushed forward the development of new drugs, as well as studies of repurposed and previously described less effective drugs [8]. The isoniazid analogues ethionamide and prothionamide were discovered in 1950-x and are in limited use in tuberculosis treatment [9,10] being included in group C of the WHO recommended drugs for the treatment of rifampicin-resistant and multidrug-resistant tuberculosis [3]. On the other hand, the gamma isomer of nicotinaldehyde thiosemicarbazone gave birth to a recently approved drug perchlozone or thioureidominomethylpyridinium perchlorate. It received official approval in November 2012 and was included in the Russian national clinical guidelines as a treatment for patients with multidrug-resistant and extensively drug-resistant tuberculosis [11,12].

**Figure 1:**
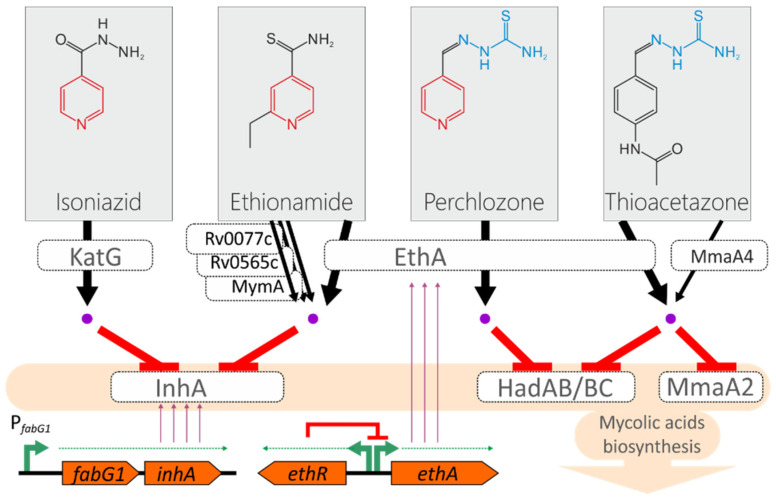
Model of the action of isoniazid, ethionamide, perchlozone, and thioacetazone on *M. tuberculosis*. The pyridine and thiosemicarbazone groups are marked with red and blue, respectively. Active forms of the drugs are shown as purple dots. The transcription of the *inhA* gene is driven by the promoter located upstream of the *fabG1* gene. The *ethA* gene that encodes monooxygenase is under transcriptional regulation by the EthR repressor.

Thioacetazone, isoniazid, ethionamide, and perchlozone have a similar mode of action and partially share the biotransformation pathways (Figure 1). They undergo intracellular conversion to toxic adducts with NAD, which target the essential enzymes of mycolic acid biosynthesis, the building blocks of the cell wall specific to mycobacteria [13]. The main target of isoniazid and ethionamide is the FAS-II cycle enzyme enoyl-acyl carrier protein (ACP) reductase, encoded by the *inhA* gene [13,14]. Perchlozone and thioacetazone act on the beta-hydroxyacyl-ACP dehydratase step of the cycle, encoded in the gene cluster *hadA-hadB-hadC* [15,16]. Thioacetazone also acts on the methoxy mycolic acid synthase MmaA2 responsible for the creation of cyclopropanated methoxymycolates [17,18].

Isonaizid as a prodrug is converted to a toxic adduct with the main katalase-peroxidase KatG, and the most frequent substitution found in resistant strains is the substitution S315T that provides the perfect balance between diminished activity towards isoniazid and retains the main activity [13]. The main enzyme that converts the ethionamide to active adducts with NADH is Baeyer–Villiger monooxygenase EthA [19,20,21]. Alternatively, it could be activated by the monooxygenases MymA, Rv0565c, and Rv077c [22].

The activation path by EthA monooxygenase is shared by thioacetazone, ethionamide, and the cross-resistance between these three drugs by loss-of-function mutations at the *ethA* locus has also been proposed [16]. Subsequently, a mutation presumably associated with resistance to thioacetazone was found in the *mmaA4* gene, which is supposed to be the alternative activator of the drug [23,24]. However, this is questioned by other researchers [25]. In addition to common activation pathways, the overexpression of the target gene *inhA* leads to the titration of isoniazid and ethionamide and therefore to cross-resistance (Figure 1) [26].

In contrast to the well-studied isoniazid and ethionamide, only a few studies have been performed on perchlozone MICs for clinical strains of *M. tuberculosis* and their molecular mechanisms of action [27]. In this report, we describe the phenotypic and genetic characteristics of clinical isolates of *M. tuberculosis* from the Moscow region, with variable susceptibility to perchlozone, ethionamide, and isoniazid. Combining phenotypic and molecular data, we estimate the proportion of drug-resistant isolates suitable for perchlozone treatment.

## 2. Results

In this study, clinical isolates from 22 patients were analyzed. Samples were obtained in 2017–2018 from patients who attended the Moscow Research and Clinical Center for Tuberculosis Control, Moscow, Russian Federation. Eight of the 22 patients had a susceptible form of tuberculosis, 12 patients had MDR-TB, and two had pre-XDR-TB, determined as additional resistance to fluoroquinolones (Table S1).

The isolates were genotyped using the 24-loci MIRU-VNTR method, and all samples belonged to the L2 Beijing lineage. Most of the isolates were from modern clonal complexes CC1 and CC2 (Table S1) [28]. Seven isolates from the Central Asian/Russian Beijing group 94-32 were predominately susceptible, while six isolates from the Beijing B0/W-148 group (MIRU type 100-32) were MDR of pre-XDR.

Resistance to ethionamide and high MICs of perchlozone were identified only in MDR isolates. The observed MIC of perchlozone ranged from 2 to 64 mg/L, with a statistically insignificant difference in the number of isolates at each concentration.

**Figure 2:**
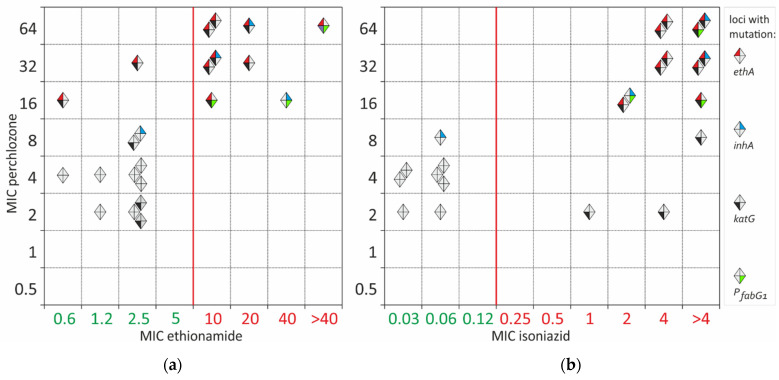
Pairwise MIC correlation of perchlozone with ethionamide (**a**) and perchlozone with isoniazid (**b**). Each isolate is shown with a diamond. Isolates with wild-type sequences are grey. Isolates with mutations are color-labelled, as indicated in the legend: mutations in *ethA* are labelled with red color of the left-top sector. Isolates with mutations in *inhA* are labelled with a blue color of the top-right sector. Isolates with mutations in *katG* are marked with black and those with mutations in the *fabG1* promoter have a green label in the bottom left and bottom right sectors, respectively.

The common activation path of perchlozone and ethionamide by EthA leads to at least partial cross-resistance between these drugs (Figure 1). All ethionamide-resistant isolates also had a high perchlozone MIC (Figure 2a). Most of the resistant isolates had an altered *ethA* sequence. However, two samples with mutations in *ethA* and a high MIC of perchlozone (16 and 32 mg/L) retained a low MIC of ethionamide of 0.6 and 2.5 mg/L, which were below the breakpoint. One of them had a substitution L440P in EthA with an unknown effect on protein function, while the other had a frameshift mutation at nucleotide position 880, which lead to the loss of EthA function. The latter strain had discordant results of resistance to ethionamide obtained by two methods—it was resistant as determined by critical concentration at 5 mg/L.

The pairwise correlation of isoniazid and perchlozone MIC revealed a partial cross-correlation (Figure 2b). Isoniazid resistance could be explained by KatG S315T substitution in 13 of 14 isolates. Only one resistant isolate had the P*_fabG1_* mutation and InhA I194V substitution. Two isolates resistant to isoniazid and presumably susceptible to perchlozone had a perchlozone MIC of 2 mg/L. No other mutations were found in addition to KatG S315T.

Two isolates had an ‘intermediate’ perchlozone MIC of 8 mg/L. One was susceptible to isoniazid and had a substitution of F97L in InhA. It was not previously described in resistant isolates and could be neutral. The second was resistant to isoniazid and had S315T substitution in KatG.

There were some discrepancies between *ethA* mutations and phenotype for three isolates with perchlozone MIC of 16mg/L. A strain had no mutations in the *ethA* gene, and high MICs of ethionamide and isoniazid could be explained by the frequently identified promoter P*_fabG1_* mutation c(-15)t and the InhA substitution I194V. Additional sequencing of the *hadABC* locus did not reveal any mutations; thus, high perchlozone MIC is caused by alterations in other genomic loci.

Nine presumable susceptible to perchlozone isolates with MICs of 2–4 mg/L had no mutations in *ethA*. They are also characterized by low MICs for ethionamides ranging from 0.6 to 2.5 mg/L (Figure 2). The H37Rv control strain of *M. tuberculosis* had a perchlozone MIC of 4 mg/L.

## 3. Discussion

Treatment of MDR-TB is challenging due to the limited number of effective drugs available and serious adverse drug reactions. Furthermore, cross-resistance between antituberculosis drugs may limit their efficacy. In addition to a common target of action for structurally similar drugs, such as in the rifamycin or fluoroquinolone groups, cross-resistance mechanisms may be due to common pathways of drug efflux or prodrug activation. For example, mutations in the transcriptional repressor gene *rv0678* lead to cross-resistance to bedaquiline and clofazimine due to derepression of the *mmpL5*-*mmpS5* efflux pump operon [29]. Even more frightening is a recent report that the same type of mutations may also lead to low-level resistance to promising new drugs from the benzothiazinone group PBTZ169 and BTZ043 [30].

Perchlozone is a relatively new drug approved and has been used in the Russian Federation since 2012. Being a member of the thiosemicarbazone family, it has a mechanism of action similar to that of thioacetozone according to experiments on in vitro selection of resistant mutants [16]. Perchlozone is a prodrug and is activated by EthA monooxygenase also as ethionamide, thioacetazone, and isoxyl [19]; therefore, cross-resistance between the currently used in tuberculosis treatment perchlozone and ethionamide has been suggested [16].

We found a wide variation in the MIC of perchlozone from 2 to 64 mg/L, correlating with the general resistance profile of the unexposed clinical strain: the MIC was higher for MDR and pre-XDR isolates. For presumably susceptible wild-type strains, the MIC of perchlozone was 2–4 mg/L. Gopal and Dick reported the perchlozone MIC_50_ value measured by optical density in 7H9 liquid medium equal to 0.04 mg/L for *M. tuberculosis* and 3.6 mg/L for *M. bovis* BCG. These values are underestimated compared to the MIC defined in the standard way [16]. Data obtained in the same study for agar medium were based on 99% growth inhibition and for *M. bovis*, the MIC of perchlozone was 18.2 mg/L. In an in vitro study of thioacetazone analogues, the MIC of a structurally matched compound (ID = 7) for the *M. tuberculosis* strain H37Rv was 0.8 mg/L, which is comparable to our data [17]. It should be noted that perchlozone was 20 times less effective than thioacetazone in the Gopal study [16] and eight times less effective in the Alahari study [17]. Average plasma perchlozone concentration at doses ranging from 800 to 1600 mg/day for patients with different body weights was 23.4 mg/L [31]. Thus, the preliminary breakpoint concentration of perchlozone in Middlebrook 7H10 agar medium could be estimated at 4–8 mg/L. This value is higher than currently approved critical concentrations of first-line rifampicin (1 mg/L) and isoniazid (0.2 mg/L), but is close to that of ethambutol—5 mg/L and kanamycin—4 mg/L [32].

In our work, we showed the cross-resistance between ethionamide and perchlozone in primary non-exposed to perchlozone *M. tuberculosis* clinical isolates. Resistance-associated mutations were located in the gene encoding EthA monooxygenase, and all strains with a high MIC of ethionamide also had a high MIC of perchlozone. A previous study on a set of clinical isolates lacked phenotypic data on the susceptibility of perchlozone, limiting the interpretation of the results [33]. However, in one case, in accordance with the proposal by Gopal and Dick and our findings, an additional frameshift mutation in the *ethA* gene emerged during perchlozone treatment. It could be proposed that isolates with elevated MIC of ethionamide and frameshift mutations in the *ethA* gene should be considered resistant, and such cases should not be treated with perchlozone.

The incomplete cross-resistance observed between isoniazid and perchlozone was due to the properties of the strains chosen for the study, since all strains with high MIC of isoniazid and perchlozone carry mutations in *ethA* and were resistant to ethionamide. Generally, susceptibility to perchlozone was retained for a part of the MDR strains, also as susceptibility to ethionamide [13,34,35], providing the possibility of therapy for such cases based on phenotypic or molecular analysis.

Other mutations could be the cause of elevated perchlozone MIC. We analyzed the HadABC locus sequence in an isolate with an MIC of 8 mg/L and wild-type *ethA*, but it was also unmutated. Another possible mechanism could be the structural similarity of perchlozone and thioacetazone. Alahari, 2007 showed the effect of thioacetazone on the family of cyclopropane mycolic acid synthases of mycobacterial enzymes responsible for the synthesis of different subtypes of mycolic acids. In the proposed model, thioacetozone inhibits MmaA2 and is activated by MmaA4 [17,24], which are involved in the production of mycolic keto acids. An independent confirmation was obtained by in vitro selection of resistant *M. tuberculosis* strains, and the obtained strains had mutations in *hadA*, *hadC*, and *mmaA4* [23].

A limitation of this study is the relatively small sample size of 22 isolates. Nevertheless, this is the first study of clinical *M. tuberculosis* isolates that combined phenotypic and molecular data and confirmed the basic assumptions about the mechanisms of resistance to perchlozone.

## 4. Materials and Methods

### 4.1. Mycobacterium tuberculosis Strains

The *M. tuberculosis* isolates were obtained from clinical specimens collected from patients with TB at the Moscow Research and Clinical Center for Tuberculosis Control, Moscow, Russian Federation. All clinical strains were isolated in 2017–2018 from sputum from newly diagnosed patients prior to starting antituberculosis therapy or no more than one month after the initiation of treatment. The laboratory reference strain *M. tuberculosis* H37Rv was used as a control strain. For molecular analysis, samples were isolated sequentially, one isolate from one patient, before initiation of treatment. Drug susceptibility tests for rifampicin, isoniazid, streptomycin, ethambutol, pyrazinamide, ofloxacin, moxifloxacin, kanamycin, capreomycin, and amikacin, PAS, and ethionamide were performed using Bactec MGIT 960 as previously described [36,37]. The detection of the MIC was performed on MYCOTB microdilution plates as described in [38]. The MIC of perchlozone was determined using serial dilution method on Middlerook 7H10 agar plates supplemented with the drug at the following concentrations: 0.5, 1, 2, 4, 8, 16, 32, and 64 mg/L.

### 4.2. DNA Isolation and Sequencing

The DNA isolation and sequencing of the *ethA*, *inhA,* and P*fabG1* fragments were performed as previously described [35]. The following PCR primers were used: ethAR-F1: 5′-cgacgttgaaatcacgctgg-3′, ethAR-R1: 5′-gtgaccgacaccattgaacg-3′; ethAR-F2: 5′-ttcaaccccgttgcggtaat-3′; ethAR-R2: 5′-ctctttctgtgcagcggcta-3′; ethAR-F3: 5′-atgatcggcccgacgaaatc-3′; ethAR-R3: 5′-ccctggcagcttactacgtg-3′; PfabG1-F: 5′-cctcgctgcccagaaaggga-3′; PfabG1-R: 5′-atcccccggtttcctccggt-3′; inhA-F2: 5′-gagctatatctccggtgcgg-3′; inhA-R2: 5′-gcgaccgtcatccagttgta-3′; inhA-F3: 5′-ccacatctcggcgtattcgt-3′; and inhA-R3: 5′-cggtgataccccaccgaaat-3′.

### 4.3. MIRU-VNTR Typing

Twenty-four loci MIRU-VNTR typing was made according to [39]. The profiles in the article are given in the following order: MIRU04(ETRD-1), MIRU26, MIRU40, MIRU10, MIRU16, MIRU31(ETRE), Mtub04, ETRC, ETR-A, Mtub30, Mtub39, Qub4156, Qub11b, Mtub21, Qub26, MIRU02, MIRU23, MIRU39, MIRU20, MIRU24, MIRU27 (Qub5), Mtub29, ETRB, Mtub34. MLVA types were compared to MIRU-VNTR*plus* online (http://www.miru-vntrplus.org/, last accessed on 12 December 2022) [40].

## Data Availability

Data are contained within the article or Supplementary Material.

## Supplementary Materials

The following supporting information can be downloaded at: https://www.mdpi.com/article/10.3390/antibiotics12030590/s1, Table S1: Summary of characterized clinical *M. tuberculosis* isolates.

## References

[1] Domagk G. (1950). Investigations on the Antituberculous Activity of the Thiosemicarbazones In Vitro and In Vivo. Am. Rev. Tuberc..

[2] Harland R.D. (1962). Stevens-Johnson Syndrome with Unusual Skin Features Occurring in Two Patients Undergoing Treatment for Pulmonary Tuberculosis with Thiacetazone. Tubercle.

[3] Jaju M., Jaju M., Ahuja Y.R. (1984). Combined and Individual Effects of Isoniazid and Thiacetazone on Human Lymphocyte Chromosomes In Vitro and In Vivo. Hum. Toxicol..

[4] Fox H.H. (1952). Synthetic Tuberculostats. III. Isonicotinaldehyde Thiosemicarbazone And Some Related Compounds. J. Org. Chem..

[5] Fox H.H. (1953). The Chemical Attack on Tuberculosis. Trans. N. Y. Acad. Sci..

[6] Stadler E.T., Steiner M. (1971). Isoniazid Treatment for Tuberculosis. N. Engl. J. Med..

[7] Parekh M.J., Schluger N.W. (2013). Treatment of Latent Tuberculosis Infection. Ther. Adv. Respir. Dis..

[8] Barry C.E., Slayden R.A., Sampson A.E., Lee R.E. (2000). Use of Genomics and Combinatorial Chemistry in the Development of New Antimycobacterial Drugs. Biochem. Pharmacol..

[9] Scardigli A., Caminero J.A., Sotgiu G., Centis R., D’Ambrosio L., Migliori G.B. (2016). Efficacy and Tolerability of Ethionamide versus Prothionamide: A Systematic Review. Eur. Respir. J..

[10] Rist N., Grumbach F., Libermann D. (1959). Experiments on the Antituberculous Activity of Alpha-Ethylthioisonicotinamide. Am. Rev. Tuberc. Pulm. Dis..

[11] Ministry of Health of the Russian Federation Russian National Guidelines on Tuberculosis Treatment. https://cr.minzdrav.gov.ru/recomend/16_2.

[12] Ministry of Health of the Russian Federation Russian National Catalog of Registered Drugs. http://zdravmedinform.ru/grls/reg-lp-001899.html.

[13] Vilchèze C., Jacobs W.R. (2014). Resistance to Isoniazid and Ethionamide in Mycobacterium Tuberculosis: Genes, Mutations, and Causalities. Microbiol. Spectr..

[14] Banerjee A., Dubnau E., Quemard A., Balasubramanian V., Um K.S., Wilson T., Collins D., de Lisle G., Jacobs W.R. (1994). InhA, a Gene Encoding a Target for Isoniazid and Ethionamide in Mycobacterium Tuberculosis. Science.

[15] Belardinelli J.M., Morbidoni H.R. (2012). Mutations in the Essential FAS II β-Hydroxyacyl ACP Dehydratase Complex Confer Resistance to Thiacetazone in Mycobacterium Tuberculosis and Mycobacterium Kansasii. Mol. Microbiol..

[16] Gopal P., Dick T. (2015). The New Tuberculosis Drug Perchlozone^®^ Shows Cross-Resistance with Thiacetazone. Int. J. Antimicrob. Agents.

[17] Alahari A., Trivelli X., Guérardel Y., Dover L.G., Besra G.S., Sacchettini J.C., Reynolds R.C., Coxon G.D., Kremer L. (2007). Thiacetazone, an Antitubercular Drug That Inhibits Cyclopropanation of Cell Wall Mycolic Acids in Mycobacteria. PLoS ONE.

[18] Glickman M.S. (2003). The MmaA2 Gene of Mycobacterium Tuberculosis Encodes the Distal Cyclopropane Synthase of the Alpha-Mycolic Acid. J. Biol. Chem..

[19] DeBarber A.E., Mdluli K., Bosman M., Bekker L.G., Barry C.E. (2000). Ethionamide Activation and Sensitivity in Multidrug-Resistant Mycobacterium Tuberculosis. Proc. Natl. Acad. Sci. USA.

[20] Baulard A.R., Betts J.C., Engohang-Ndong J., Quan S., McAdam R.A., Brennan P.J., Locht C., Besra G.S. (2000). Activation of the Pro-Drug Ethionamide Is Regulated in Mycobacteria. J. Biol. Chem..

[21] Vannelli T.A., Dykman A., Ortiz de Montellano P.R. (2002). The Antituberculosis Drug Ethionamide Is Activated by a Flavoprotein Monooxygenase. J. Biol. Chem..

[22] Laborde J., Deraeve C., Duhayon C., Pratviel G., Bernardes-Génisson V. (2016). Ethionamide Biomimetic Activation and an Unprecedented Mechanism for Its Conversion into Active and Non-Active Metabolites. Org. Biomol. Chem..

[23] Coxon G.D., Craig D., Corrales R.M., Vialla E., Gannoun-Zaki L., Kremer L. (2013). Synthesis, Antitubercular Activity and Mechanism of Resistance of Highly Effective Thiacetazone Analogues. PLoS ONE.

[24] Alahari A., Alibaud L., Trivelli X., Gupta R., Lamichhane G., Reynolds R.C., Bishai W.R., Guerardel Y., Kremer L. (2009). Mycolic Acid Methyltransferase, MmaA4, Is Necessary for Thiacetazone Susceptibility in Mycobacterium Tuberculosis. Mol. Microbiol..

[25] Belardinelli J.M., Morbidoni H.R. (2013). Recycling and Refurbishing Old Antitubercular Drugs: The Encouraging Case of Inhibitors of Mycolic Acid Biosynthesis. Expert Rev. Anti Infect. Ther..

[26] Rueda J., Realpe T., Mejia G.I., Zapata E., Rozo J.C., Ferro B.E., Robledo J. (2015). Genotypic Analysis of Genes Associated with Independent Resistance and Cross-Resistance to Isoniazid and Ethionamide in Mycobacterium Tuberculosis Clinical Isolates. Antimicrob. Agents Chemother..

[27] Malík I., Čižmárik J., Pecháčová M. (2020). Focus on Perchlozone, an Anti-Tuberculosis Drug from the Russian Federation. Ceska Slov. Farm..

[28] Merker M., Blin C., Mona S., Duforet-Frebourg N., Lecher S., Willery E., Blum M.G.B., Rüsch-Gerdes S., Mokrousov I., Aleksic E. (2015). Evolutionary History and Global Spread of the Mycobacterium Tuberculosis Beijing Lineage. Nat. Genet..

[29] Hartkoorn R.C., Uplekar S., Cole S.T. (2014). Cross-Resistance between Clofazimine and Bedaquiline through Upregulation of MmpL5 in Mycobacterium Tuberculosis. Antimicrob. Agents Chemother..

[30] Poulton N.C., Azadian Z.A., DeJesus M.A., Rock J.M. (2022). Mutations in Rv0678 Confer Low-Level Resistance to Benzothiazinone DprE1 Inhibitors in Mycobacterium Tuberculosis. Antimicrob. Agents Chemother..

[31] Yablonskiy P.K., Vinogradova T.I., Levashev Y.N., Pavlova M.V., Zilber E.K., Starshinova A.A., Sapozhnikova N.V., Chernokhaeva I.V., Archakova L.I., Zabolotnykh N.V. (2016). Preclinical and Clinical Trials of the New Tuberculosis Drug Perchlozon. Ter. Arkhiv.

[32] WHO (2018). Technical Report on Critical Concentrations for Drug Susceptibility Testing of Medicines Used in the Treatment of Drug-Resistant Tuberculosis.

[33] Mokrousov I., Vyazovaya A., Akhmedova G., Solovieva N., Turkin E., Zhuravlev V. (2020). Genetic Variation Putatively Associated with Mycobacterium Tuberculosis Resistance to Perchlozone, a New Thiosemicarbazone: Clues from Whole Genome Sequencing and Implications for Treatment of Multidrug-Resistant Tuberculosis. Antibiotics.

[34] Morlock G.P., Metchock B., Sikes D., Crawford J.T., Cooksey R.C. (2003). EthA, InhA, and KatG Loci of Ethionamide-Resistant Clinical Mycobacterium Tuberculosis Isolates. Antimicrob. Agents Chemother..

[35] Ushtanit A., Kulagina E., Mikhailova Y., Makarova M., Safonova S., Zimenkov D. (2022). Molecular Determinants of Ethionamide Resistance in Clinical Isolates of Mycobacterium Tuberculosis. Antibiotics.

[36] Sharma M., Thibert L., Chedore P., Shandro C., Jamieson F., Tyrrell G., Christianson S., Soualhine H., Wolfe J. (2011). Canadian Multicenter Laboratory Study for Standardized Second-Line Antimicrobial Susceptibility Testing of Mycobacterium Tuberculosis. J. Clin. Microbiol..

[37] Barrera L., Cooreman E., de Dieu Iragena J., Drobniewski F., Duda P., Havelkova M., Hoffner S., Kam K.M., Kim S.J., Labelle S. (2008). Policy Guidance on Drug-Susceptibility Testing (DST) of Second-Line Antituberculosis Drugs.

[38] Nosova E.Y., Zimenkov D.V., Khakhalina A.A., Isakova A.I., Krylova L.Y., Makarova M.V., Galkina K.Y., Krasnova M.A., Safonova S.G., Litvinov V.I. (2016). A Comparison of the Sensititre MycoTB Plate, the Bactec MGIT 960, and a Microarray-Based Molecular Assay for the Detection of Drug Resistance in Clinical Mycobacterium Tuberculosis Isolates in Moscow, Russia. PLoS ONE.

[39] Supply P., Allix C., Lesjean S., Cardoso-Oelemann M., Rüsch-Gerdes S., Willery E., Savine E., de Haas P., van Deutekom H., Roring S. (2006). Proposal for Standardization of Optimized Mycobacterial Interspersed Repetitive Unit-Variable-Number Tandem Repeat Typing of Mycobacterium Tuberculosis. J. Clin. Microbiol..

[40] Allix-Béguec C., Harmsen D., Weniger T., Supply P., Niemann S. (2008). Evaluation and Strategy for Use of MIRU-VNTRplus, a Multifunctional Database for Online Analysis of Genotyping Data and Phylogenetic Identification of Mycobacterium Tuberculosis Complex Isolates. J. Clin. Microbiol..

