# Supplementary material for: Perchlozone Resistance in Clinical Isolates of Mycobacterium tuberculosis
Source: Antibiotics (Basel). 2023 Mar 15;12(3):590. doi: 10.3390/antibiotics12030590 (PMC10044601; doi:10.3390/antibiotics12030590)
Supplement: Supplementary file 1 [file antibiotics-12-00590-s001.zip › antibiotics-2189360-supplementary.pdf]

Table S1: Summary of characterized clinical *M. tuberculosis* isolates.

ID	Resistance profile	Isoniazid		Ethionamide		Perchlozone MIC, 7H11	Mutations				Beijing clonal complex	MLVA type
		Bactec	MIC	Bactec	MIC		*ethA*	*P_fabG1*	*inhA*	*katG*		
7	S	S	0.06	S	2.5	4	wt	wt	wt	wt	CC1	94-32
13	S	S	0.06	S	2.5	2	wt	wt	wt	wt	CC1	94-32
14	S	S	0.03	S	1.2	2	wt	wt	wt	wt	CC1	94-32
15	S	S	0.03	S	1.2	4	wt	wt	wt	wt	CC1	94-32
22	S	S	0.06	S	2.5	8	wt	wt	g291a F97L	wt	CC1	94-32
18	MDR	R	>4	S	2.5	8	wt	wt	wt	S315T	CC1	94-32
8	MDR	R	>4	R	>40	64	t276tggaa	t(-8)g	wt	S315T	CC1	94-32
19	S	S	0.06	S	2.5	4	wt	wt	wt	wt	CC1	95-32
21	S	S	0.06	S	2.5	4	wt	wt	wt	wt	CC1	99-32
2	MDR	R	4	R	10	64	t(-6)c	wt	wt	S315T	CC1	1065-32
11	MDR	R	>4	R	10	32	t(-7)c	wt	g707a T236K	S315T	CC1	1065-32
5	MDR	R	2	R	40	16	wt	c(-15)t	t589c I194V	wt	CC1	9136-32
1	MDR	R	4	S	2.5	2	wt	wt	wt	S315T	CC2	100-32
3	MDR	R	2	S	0.6	16	t1319c L440P	wt	wt	S315T	CC2	100-32
6	MDR	R	4	R	10	64	g(-55)a	wt	wt	S315T	CC2	100-32
9	pre-XDR	R	4	R	20	32	ga106g	wt	wt	S315T	CC2	100-32
10	MDR	R	>4	R	10	16	ga337g	c(-34)t	wt	S315T	CC2	100-32
12	pre-XDR	R	>4	R	10	32	ga106g	wt	wt	S315T	CC2	100-32
4	MDR	R	1	S	2.5	2	wt	wt	wt	S315T	CC2	9375-32
20	MDR	R	>4	R	20	64	ct702c	wt	g535a A179T	S315T	CC2	5317-32
16	S	S	0.03	S	0.6	4	wt	wt	wt	wt	CC3	1068-32
17	MDR	R	4	R	2.5	32	ct882c	wt	wt	S315T	CC3	1066-32

ID	MIRU-VNTR																							
	MIRU04(ETRD-1)	MIRU26	MIRU40	MIRU10	MIRU16	MIRU31(ETRE)	Mtub04	ETRC	ETR-A	Mtub30	Mtub39	Qub4156	Qub11b	Mtub21	Qub26	MIRU02	MIRU23	MIRU39	MIRU20	MIRU24	MIRU27 (Qub5)	Mtub29	ETRB	Mtub34
7	2	5	3	3	3	5	4	4	4	4	3	2	6	5	8	2	5	3	2	1	3	4	2	3
13	2	5	3	3	3	5	4	4	4	4	3	2	6	5	8	2	5	3	2	1	3	4	2	3
14	2	5	3	3	3	5	4	4	4	4	3	2	6	5	8	5	5	3	2	1	3	4	2	3
15	2	5	3	3	3	5	4	4	4	4	3	2	6	5	8	5	5	3	2	1	3	4	2	3
22	2	5	3	3	3	5	4	4	4	4	3	2	6	5	8	2	5	3	2	1	3	4	2	3
18	2	5	3	3	3	5	4	4	4	4	3	2	6	5	8	2	5	3	2	1	3	4	2	3
8	2	5	3	3	3	5	4	4	4	4	3	2	6	5	8	2	5	3	2	1	3	4	2	3
19	2	5	3	3	3	5	4	4	3	4	3	2	6	5	8	2	5	3	2	1	3	4	2	3
21	2	5	3	3	3	5	4	4	3	4	4	2	6	5	7	2	5	3	2	1	3	4	2	3
2	2	5	3	3	3	5	4	4	4	4	3	2	6	5	6	2	5	3	2	1	3	4	2	3
11	2	5	3	3	3	5	4	4	4	4	3	2	6	5	6	2	5	3	2	1	3	4	2	3
5	2	5	4	3	3	4	4	4	4	4	3	2	1	5	7	2	5	3	2	1	3	4	2	3
1	2	7	3	3	3	5	4	4	4	4	3	2	6	5	7	2	5	3	2	1	3	4	2	3
3	2	7	3	3	3	5	4	4	4	4	3	2	6	5	7	2	5	3	2	1	3	4	2	3
6	2	7	3	3	3	5	4	4	4	4	3	2	6	5	7	2	5	3	2	1	3	4	2	3
9	2	7	3	3	3	5	4	4	4	4	3	2	6	5	7	2	5	3	2	1	3	4	2	3
10	2	7	3	3	3	5	4	4	4	4	3	2	6	5	7	2	5	3	2	1	3	4	2	3
12	2	7	3	3	3	5	4	4	4	4	3	2	6	5	7	2	5	3	2	1	3	4	2	3
4	2	7	4	3	3	5	4	4	4	4	3	2	6	5	7	2	5	3	2	1	3	4	2	3
20	2	7	3	3	3	2	4	4	4	4	3	2	6	5	7	2	5	3	2	1	3	4	2	3
16	2	4	3	3	3	5	4	4	4	4	3	2	6	5	8	5	5	3	2	1	3	4	2	3
17	2	7	3	3	3	4	4	4	4	4	3	2	6	5	6	5	5	3	2	1	3	4	2	3
